# Understanding Changes in Physical Activity among Lower Limb Prosthesis Users: A COVID‐19 Case Series

**DOI:** 10.1002/pmrj.12508

**Published:** 2020-12-04

**Authors:** Akshay S. Tolani, Shane R. Wurdeman, Bryce Billing, Erin O'Brien, Dwiesha L. England, Phillip M. Stevens, Aaron Flores

**Affiliations:** ^1^ Department of Clinical and Scientific Affairs Hanger Clinic Austin TX USA; ^2^ Department of Information Technology Hanger Clinic Austin TX USA; ^3^ Hanger Fabrication Network Hanger Clinic Austin TX USA

The coronavirus disease 2019 (COVID‐19) pandemic has created pressures for social distancing and avoidance of large gatherings as well as multiple states declaring shutdowns in March 2020.^1^ The result is pressure for individuals to only leave their homes for necessities with reduced frequency. This creates potential for reduced physical activity, which is problematic for patients with limb loss.^2^, ^3^, ^4^, ^5^

Hanger Inc. (Austin, TX, USA) has recently developed activity monitors that directly attach to an individual's prosthetic leg (EmpowerGO, Hanger Inc., Austin, TX, USA). The unique characteristic of the EmpowerGO step activity monitor (SAM) is its ability to remotely transmit activity data directly to a third party such as the individual's prosthetist or physician, independent of any smartphone or computer. At the time of the emergence of the COVID‐19 pandemic, Hanger Clinic was in the process of testing the SAM. Devices were being worn on a handful of individuals to assess potential opportunities for design improvement (eg, size of device, look of device, battery fatigue, and so on). Once the pandemic emerged, the individuals continued wearing the devices until they were able to return to the clinic. As a result, these individuals wearing the devices have provided a unique ability to understand the potential changes to physical activity for individuals with lower limb amputation during shutdown and “shelter‐in‐place” orders.

The SAM device was designed through collaboration between Hanger Inc. and AT&T (Dallas, TX, USA), with dynamic learning algorithms developed and tested specific to the population of lower limb prosthesis users to calculate step counts, step bouts, average time per step bout, and average steps per bout. To establish step count fidelity, a single individual, Person 0, wore the unit from June 28, 2019 through January 1, 2020, prior to COVID‐19. Person 0 also wore a microprocessor knee (MPK) with a built‐in step count function. Note patient demographics in online Appendix A. It was determined a priori that an agreement between the MPK step count and the SAM step count within 10% would be considered acceptable. From July 18, 2019 through December 19, 2019, a total 155 days, the MPK reported a total of 559 046 steps versus 509 594 for the SAM, for a discrepancy of 49 452 steps, or 8.85%. Calculation of average steps per day measured by the MPK equated to 3606.7, which is 319.0 steps more than the average of 3287.7 measured by SAM during this window. However, for the SAM there were 25 days during this period in which a block of delayed data transmission exceeded 5 hours, compromising the integrity of the daily activity report. When analysis was confined to the 130 days without delayed data transmission, a step count of 486 234 was recorded, yielding an average of 3740.3 daily steps, or 133.6 steps more than recorded by the MPK, for a discrepancy of −3.70%.

For the three individuals monitored during the initiation of COVID‐19, Person 2 had the highest overall average daily step count, followed by Person 1, Person 0, and Person 3 (Table 1). In terms of absolute daily step count variability, Person 1 also had the greatest with a standard deviation of 3288.9. In terms of relative daily step count variability, Person 3 had the highest coefficient of variation at 52.2%. The day‐to‐day activity before and after the arbitrary index point of the control case shows that Person 0 demonstrated no general variation in activity as expected beyond the day‐to‐day step count variability of 1573.5 steps (Figure 1). Person 1 actually increased activity up until about day 40 after the index date, but then declined back to activity levels similar to those previous. Person 2 and Person 3, however, experienced a notable decline in step activity. When each individual's step activity was normalized to their step activity pre‐index, the decline in step activity is further observed for Persons 2 and 3, with Person 2 reducing daily step count by nearly 6000 steps from pre‐index (online Appendix C‐ Figure 1). Similar findings were observed with step bouts, average steps per bout, and average time per step (see online Appendix D).

**Table 1:** Daily step count including pre‐ and post‐changes from an index date of March 1, 2020

		Person 0			Person 1			Person 2			Person 3	
	Average	SD	CoV	Average	SD	CoV	Average	SD	CoV	Average	SD	Car
Overall	3724.7	1573.5	42.2	5010.6	1215.1	24.2	8000.6	3288.9	41.1	1698.8	887.2	52.2
Pre‐Index*	3733.9	1547.5	41.4	4462.6	1229.7	27.6	9369.4	3002.3	32.0	2151.0	562.3	26.1
Post–Index*	3718.5	1598.7	43.0	5132.4	1186.1	23.1	5754.4	2399.6	41.7	911.5	798.3	87.6

SD = standard deviation; CoV = coefficient of variation; Units for average and SD: steps, CoV presented as percentage.

* Person 0 index date is arbitrary days into wearing activity monitor centered based on earliest point pre‐index worn by other individuals for presentation.

**Figure 1:**
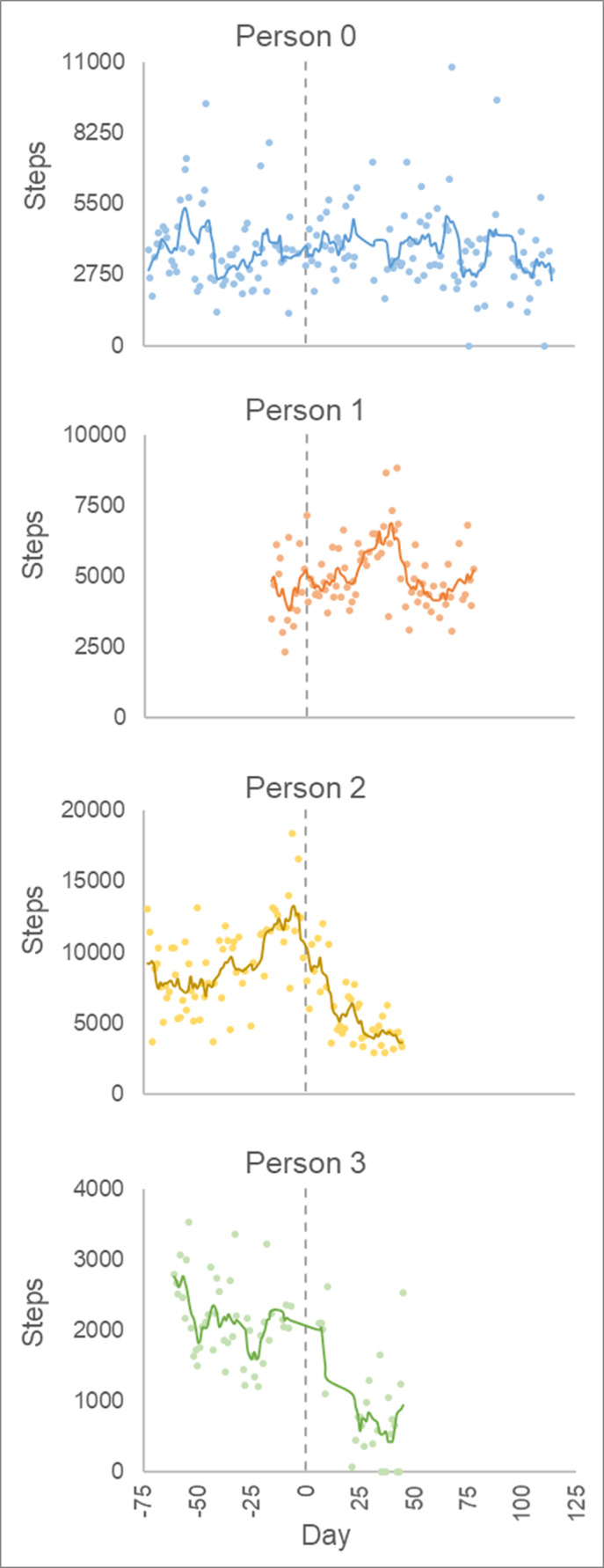
Day‐to‐day step activity data for the four subjects shows a substantial decline in step activity for Persons 2 and 3. For Persons 1 to 3, the vertical dashed line represents the index date as of March 1, 2020. For Person 0 the index date is 73 days into wearing the activity monitor, which is the longest pre‐index period of any other individuals observed through beginning of COVID‐19 pandemic. Solid line: rolling 7‐day average.

There was a decline in activity observed among two of the three individuals who were actively testing the SAM system through beginning stages of the COVID‐19 pandemic (Persons 2 and 3), whereas Person 1 demonstrated a more unique behavior. First, for Person 2 and 3, there was what would be considered a substantial decline in physical activity in terms of steps and bouts. Person 2 reduced his/her number of daily steps by ~6000 steps 45 days into the post‐index period (Figure 1). Person 3 reduced his/her steps by ~1700 steps 40 days into the post‐index period. Previous work has shown a reduction of daily steps by ~1500 steps per day can lead to a loss of 4% leg muscle mass in a 14‐day period.^6^ Furthermore, these declines represent a decline of more than ~64% (6000/9369) and ~ 79% (1700/2151) for Persons 2 and 3, respectively. This is approaching and even passes the approximate 75% reduction Reidy et al^7^ noted to drive 8% reduction in muscle strength through a 14‐day period.

During the COVID‐19 global health pandemic, there have been shelter‐in‐place and social distancing orders eliminating opportunities for routine activities. These orders have been given in an effort to control the spread of COVID‐19; however, for the millions of Americans exerting reduced physical activity, there is a real concern that these orders may be creating pressures that are resulting in more physical inactivity. There were clear signs of overall reduced activity among two of the three individuals. Health care providers involved in rehabilitation should be aware of the potential for pressures leading to reduced physical activity, whether it be external pressures from COVID‐19 or other pandemics, or internal pressures such as acute health events. Furthermore, researchers and scientists should exercise caution when interpreting results from studies incorporating step activity and physical activity as end point measures during the COVID‐19 pandemic.

## Supporting information


**Appendix** **S1**. Supporting Information
