# Supplementary material for: Understanding Changes in Physical Activity among Lower Limb Prosthesis Users: A COVID‐19 Case Series
Source: PM R. 2020 Dec 4;13(1):97–9. doi: 10.1002/pmrj.12508 (PMC7675514; doi:10.1002/pmrj.12508)
Supplement: Supplementary file 1 — Appendix S1. Supporting Information [file PMRJ-13-97-s001.docx]

**Appendix A - Demographics**

Person 0 was age 30-39, with a unilateral, transfemoral amputation due to non-dysvascular etiology (i.e. trauma, congenital, or cancer) between 3-5 years prior. This individual’s mobility, reported using the Prosthesis Limb User’s Survey of Mobility (PLUS-M), was reported at a T-score of 62.5, placing him in the 89.5% of all lower limb prosthesis users’ mobility. Person 0 was classified as a K3 level ambulator, employed through the duration of the period he wore the SAM. His employment involved greater than 50% of time standing or walking. His prosthesis utilized an ischial containment suction socket incorporating a seal-in liner system (Seal-In^®^, Össur, Reykavik, Iceland), a MPK (C-Leg, Ottobock, Dudderstadt, Germany), and a dynamic response foot (Triton, Ottobock, Dudderstadt, Germany).

Person 1 was age 40-49, with a unilateral, knee disarticulation amputation due to non-dysvascular etiology (i.e. trauma, congenital, or cancer) less than 1 year prior. The individual’s PLUS-M T-score in July 2020 was 52.7, placing Person 1 at the 60.8% for mobility. He was classified as a K3 level ambulator, employed through the duration of the period he/she wore the SAM. Prior to COVID-19, he went into an office everyday working at a desk job with less than 50% of time standing or walking. Once COVID-19 presented with shelter-in-place orders, he worked exclusively from home. His prosthesis was comprised of a subischial socket utilizing a silicone liner (Össur, Reykavik, Iceland), a MPK (C-Leg, Ottobock, Dudderstadt, Germany), and a dynamic response foot (Triton LP, Ottobock, Dudderstadt, Germany).

Person 2 was between ages 20-29, with a unilateral, transtibial amputation due to non-dysvascular etiology (i.e. trauma, congenital, or cancer) more than 10 years prior. The individual’s PLUS-M T-score in September 2019 was 67.1, placing Person 2 at the 95.6% for mobility. Person 2 was classified as a K4 level ambulator, and is a collegiate baseball player. He was employed prior to COVID-19 in the entertainment industry (e.g. party supply rentals), which required greater than 50% of time standing or walking. Limitations on large gatherings and shelter-in-place orders led to reduced work time. Furthermore, his baseball practices were subsequently cancelled with the halt of collegiate sports. His prosthesis was comprised of a hybrid patellar-tendon bearing/total-surfacing bearing interface with anatomical suspension and a pelite liner. The foot was a dynamic response foot (Cheetah Xplore, Össur, Reykavik, Iceland).

Person 3 was age 70-79, with a unilateral, transtibial amputation due to dysvascular/diabetic etiology 3-5 years prior. The individual’s PLUS-M T-score in February 2020 was 47.7, placing Person 3 at the 41.1% for mobility. Person 3 was classified as a K3 level ambulator, although noted to be lower in the spectrum of K3 from Person 0 and 1. He was retired prior to COVID-19. Clinical notes taken indicate he had the potential and ability to ambulate at variable cadences, and across various terrains, but overall participated in low levels of activity. His prosthesis was comprised of a patellar-tendon bearing interface with pelite liner, sleeve suspension, and a dynamic response foot (Maverick, Freedom Innovations, Irvine, California, United States). Person 3 also utilizes an articulated thermoplastic ankle-foot orthosis on the contralateral limb for ankle instability.

**Appendix B – Data Endpoints and Reduction**

For the three individuals monitored during the initiation of COVID-19, Person 1 had 88 days of activity data, 16 of which occurred prior to the index date of March 1, 2020. Person 2 had 103 days of activity data, 64 occurred prior to the index date. Person 3 had 74 days of activity data, 47 occurred prior to the index date.

At the time of the current case series, the SAM provided daily step count and step bout activity. A step bout was defined to be stepping activity occurring after a period of inactivity of at least 10 seconds and lasting until another period of non-stepping of at least 10 seconds. This provided two additional endpoint measures beyond step count and number of bouts per day; the average number of steps per bout and time per bout were calculated. Step bouts provide additional insight into physical activity as some individuals may move with fewer steps but more frequently through the day as opposed to someone that is fairly lethargic with exception of a single large activity bout.

Summary minute data is streamed through cellular data networks every hour unless a signal is not available in which case data is retained until such signal is available. If the device is unable to transmit data, it can retain up to five hours of data before data begins to overwrite within the onboard memory. Subsequently, when comparing step counts between the SAM and MPK for Person 0, all step data over the specified period was included. However, for subsequent analyses and presentation investigating day-to-day activity changes through the initial period of lockdown with COVID-19, days with at least one block period of delayed data transmission for five or more hours were discarded.

For understanding the impact of COVID-19 on physical activity, while the timeline varies depending on region and location, the growing pressures and awareness began in February 2020. The three individuals wearing the SAM through the beginning stages of COVID-19 were all based in Portland, Oregon, which subsequently had shelter-in-place orders enacted in March 2020. For illustrative purposes only, the timeline was indexed against March 1, 2020, subsequently denoting this as “Day 0”, with negative day numbers preceding and increasing numbers following. However, it should be noted that the development of the pandemic and subsequent potential behavior changes would have varied slightly among individuals, especially in the event of personal comfort level with risks of exposure. For Person 0, as there was no event to index against, the furthest pre-index day that was noted for any of the three individuals during COVID was used as the start point. Specifically, this was -73 for Person 2. To protect patient identity, person demographics are presented as categorical values.

There is strong support for long term activity monitoring. This case series was feasible through new technology that did not require the observed individuals to have any additional hardware or software, or to undertake additional clinic visits to download stored mobility data. The new technology was noted to have a step count agreement within the targeted 10% goal, and on days with data recorded was able to reach agreement within 5%. However, 16% 25/155 days had a 5-hour block of data missing and subsequently some activity data may have been lost on these days. Future updates will reduce these days.

**Appendix C – Step Count Normalized**

When normalizing each individual’s step activity to their step activity pre-index, the decline in step activity is further observed for Persons 2 and 3 with Person 2 reducing daily step count by nearly 6000 steps from pre-index.

Figure C2: Normalizing step activity to the mean pre-index daily step count allows observation of relative changes. In particular, this is important for cases such as Person 2 where it is observed that while after 45 days post-index the individual is still maintaining step activity similar to pre-index for the other three individuals, this activity represents a decline of nearly ~6000 steps per day. Solid line: rolling seven-day average.


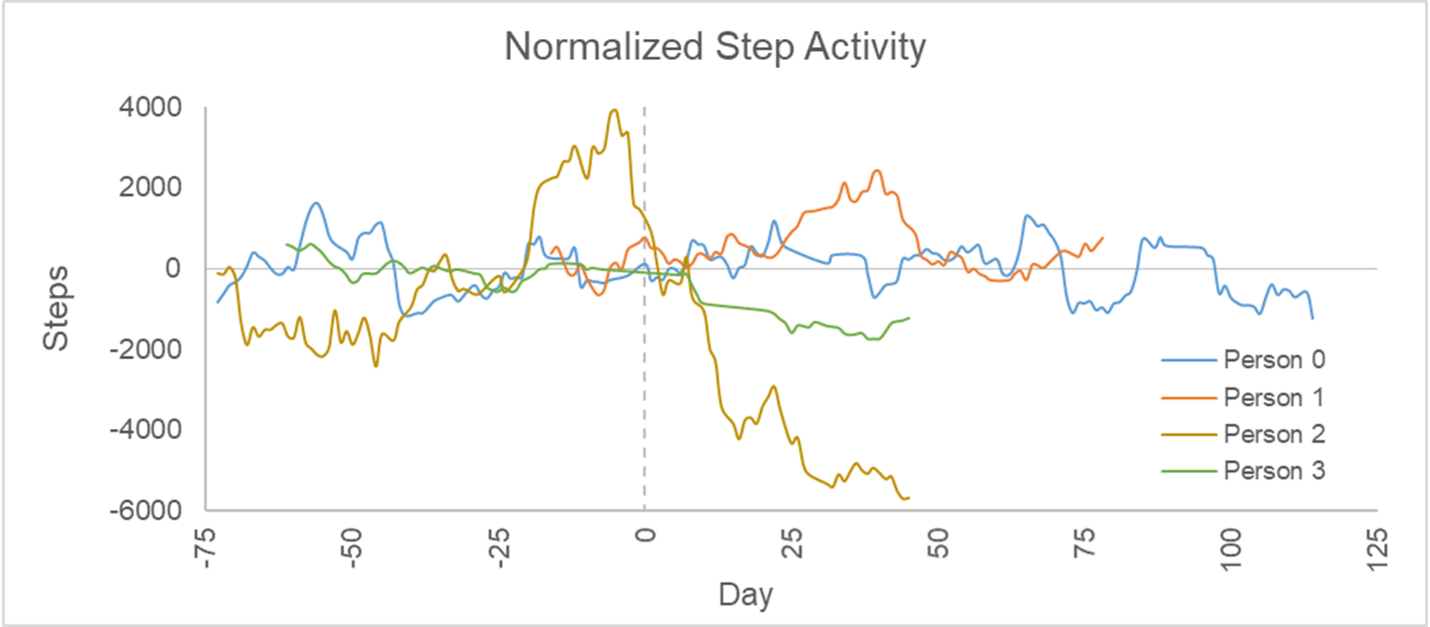


**Appendix D – Step Bouts, Average Steps per Bout, and Average Time per Bout**

*Step Bouts Through COVID-19*

Bouts of Stepping Activity

Overall, consistent with steps per day, Person 2 averaged the most bouts per day, followed by Person 1, Person 3, and lastly Person 0 (Table D2). A similar trend was noted for the pre-index period. For post-index, Person 1 had the most bouts per day with an increase over the pre-index period. This was followed by Person 2, Person 0, and lastly Person 3.

Table D2: Daily bout data, including pre- and post- changes from an index date of March 1, 2020. SD: standard deviation; CoV: coefficient of variation; *Person 0 index date is arbitrary days into wearing activity monitor centered based on earliest point pre-index worn by other individuals for presentation.

Examining the day-to-day changes in bouts, it is observed that Person 0 maintained fairly consistent behavior with regards to number of bouts (Figure D3). Person 1 had a trend of increasing bouts as days progressed past index date. Person 2 continued to reduce number of bouts as days progressed past index date. Person 3 seemingly reduced bouts ~25 days prior to index date.

Steps per Bout

For steps per bout, Person 2 again averaged the most steps per bout overall, followed by Person 0, Person 1, and finally Person 3 (Table D2). The steps per bout pre-index and post-index followed a similar trend among the individuals.

Examining day-to-day changes in steps per bout, Person 1 had a gradual decline in steps per bout as days progressed beyond index date (Figure D4). Person 2 conversely rapidly dropped the steps per bout and then tapered while Person 3 again adjusted to increased steps per bout ~25 days prior to index date.


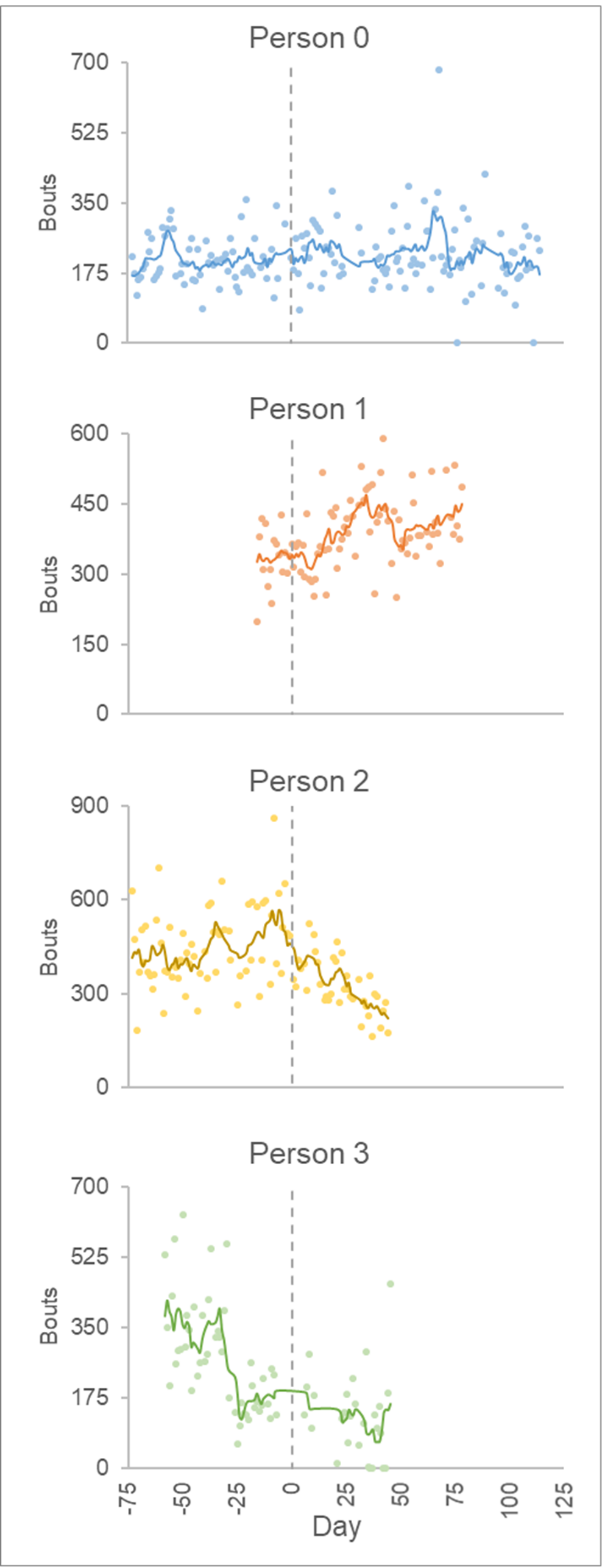


Figure D3: Day-to-day number of activity bouts for the four subjects shows a substantial decline in number of bouts for Persons 2 and 3, with Person 3 beginning reduction ~25 days prior to index day. Solid line: rolling seven-day average.


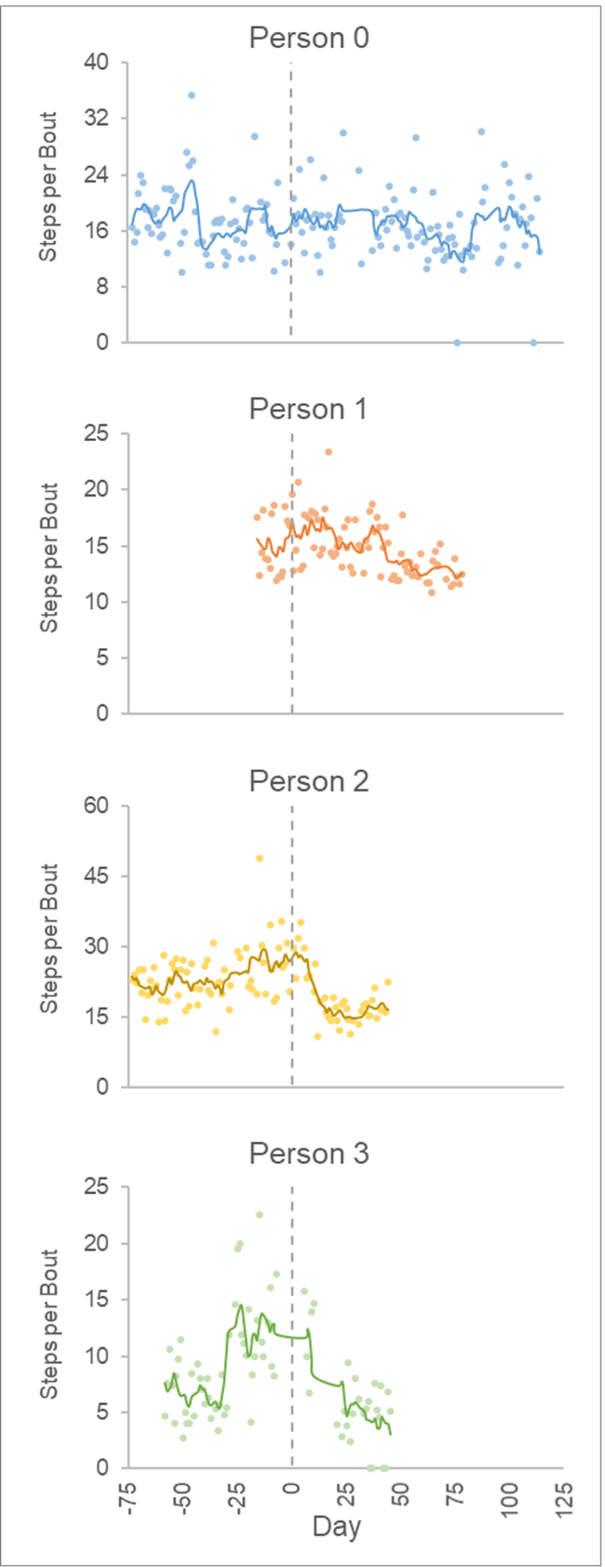


Figure D4: Day-to-day change in number of steps per bout for the four subjects shows a gradual decline in number of bouts for Person 1 and a more drastic decline in Person 2. Coinciding with Person 3 initial reduction in bouts was an increase in steps per bout beginning ~25 days prior to index day. Solid line: rolling seven-day average.

Time per Bout


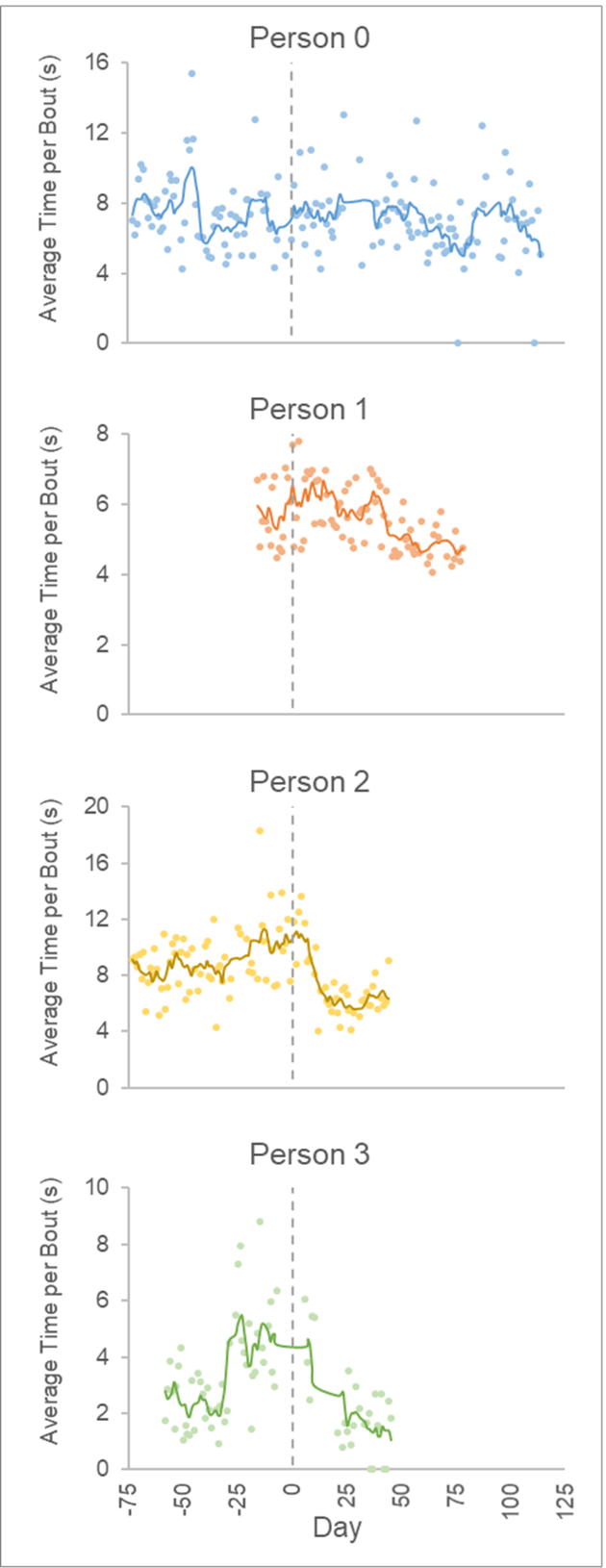


Figure D5: Day-to-day change in time per bout for the four subjects largely mirrors changes seen in steps per bout. This would seem to indicate the amount of time per step, or average cadence, remained constant through the observation period. Solid line: rolling seven-day average.

For time per bout, again Person 2 had overall the greatest time per bout, followed again by Person 0, then Person 1 and 3 (Table D2). Pre-index and post-index trends among individuals were consistent with steps per bout data. Day-to-day changes in time per bout were also similar pattern as steps per bout (Figure D5).
